# Autophagy Impairment Induces Premature Senescence in Primary Human Fibroblasts

**DOI:** 10.1371/journal.pone.0023367

**Published:** 2011-08-08

**Authors:** Hyun Tae Kang, Ki Baek Lee, Sung Young Kim, Hae Ri Choi, Sang Chul Park

**Affiliations:** 1 Department of Biochemistry and Molecular Biology, Seoul National University College of Medicine, Seoul, Korea; 2 Institute on Aging, Seoul National University College of Medicine, Seoul, Korea; 3 Lee Gil Ya Cancer and Diabetes Institute, Gachon University of Medicine and Science, Incheon, Korea; University of Minnesota, United States of America

## Abstract

**Background:**

Recent studies have demonstrated that activation of autophagy increases the lifespan of organisms from yeast to flies. In contrast to the lifespan extension effect in lower organisms, it has been reported that overexpression of unc-51-like kinase 3 (ULK3), the mammalian homolog of autophagy-specific gene 1 (ATG1), induces premature senescence in human fibroblasts. Therefore, we assessed whether the activation of autophagy would genuinely induce premature senescence in human cells.

**Methodology/Principal Findings:**

Depletion of ATG7, ATG12, or lysosomal-associated membrane protein 2 (Lamp2) by transfecting siRNA or infecting cells with a virus containing gene-specific shRNA resulted in a senescence-like state in two strains of primary human fibroblasts. Prematurely senescent cells induced by autophagy impairment exhibited the senescent phenotypes, similar to the replicatively senescent cells, such as increased senescence associated β-galactosidase (SA-β-gal) activity, reactive oxygen species (ROS) generation, and accumulation of lipofuscin. In addition, expression levels of ribosomal protein S6 kinase1 (S6K1), p-S6K1, p-S6, and eukaryotic translation initiation factor 4E (eIF4E) binding protein 1 (4E-BP1) in the mammalian target of rapamycin (mTOR) pathway and beclin-1, ATG7, ATG12-ATG5 conjugate, and the sequestosome 1 (SQSTM1/p62) monomer in the autophagy pathway were decreased in both the replicatively and the autophagy impairment-induced prematurely senescent cells. Furthermore, it was found that ROS scavenging by N-acetylcysteine (NAC) and inhibition of p53 activation by pifithrin-α or knockdown of p53 using siRNA, respectively, delayed autophagy impairment-induced premature senescence and restored the expression levels of components in the mTOR and autophagy pathways.

**Conclusion:**

Taken together, we concluded that autophagy impairment induces premature senescence through a ROS- and p53-dependent manner in primary human fibroblasts.

## Introduction

Autophagy is an evolutionarily conserved catabolic process, by which cytoplasmic proteins and organelles are engulfed in autophagosomes and degraded after fusion with lysosomes. Biological roles of autophagy can be illustrated in a variety of physiological and pathophysiological conditions, such as starvation adaptation, clearance of damaged proteins and organelles, development, elimination of pathogens, cell survival and death, tumor suppression, and antigen presentation [1]. In addition, it has been suggested that autophagy can have a pro-longevity effect in organisms from yeast to flies [2]–[6], although this hypothesis remains controversial [7]–[8]. Furthermore, several lifespan extension interventions, including calorie restriction as well as treatment with resveratrol, rapamycin, or spermidine, have exploited their effects through activation of autophagy [5], [9]–[11]. Although the roles of autophagy in age-related diseases involving neurodegenerative dysfunction, such as Alzheimer's disease, Parkinson's disease, Huntington's disease [12]–[14], macular degeneration [15]–[16], hypercholesterolemia [17], and cardiomyopathy [18], have been implicated, the effect of autophagy on the lifespan of mammals is still ambiguous. Mice with autophagy impairment by the homozygous knockout of ATG5, ATG7, Beclin-1, or Ambra1 genes revealed embryonic lethality or postnatal death [19]. Mice with a heterozygous deletion of beclin-1 showed increased signs of morbidity, including a high incidence of spontaneous tumors [20]–[21]. Moreover, deletion of the huntingtin polyglutamine tract in a huntington's disease mouse model with mutant huntingtin showed enhanced longevity that was most likely related to the activation of autophagy [22]. In contrast, overexpression of ULK3, which is an isoform of ULK1, the mammalian homolog of ATG1, enhances autophagy but results in premature senescence in human fibroblasts [23]. This equivocal result brought confusion to the precise role of autophagy in senescence. Therefore, we examined the effect of autophagy impairment on replicative lifespan using RNAi-mediated knockdown of 3 genes involved in autophagy (ATG7, ATG12, and Lamp2) in two primary human fibroblasts in order to clarify the role of autophagy during cellular senescence. In this study, we found that inhibition of autophagy can induce premature senescence in primary human diploid fibroblasts in a reactive oxygen species (ROS)- and p53-dependent manner.

## Results

### Autophagy impairment induces premature senescence

**Figure 1:**
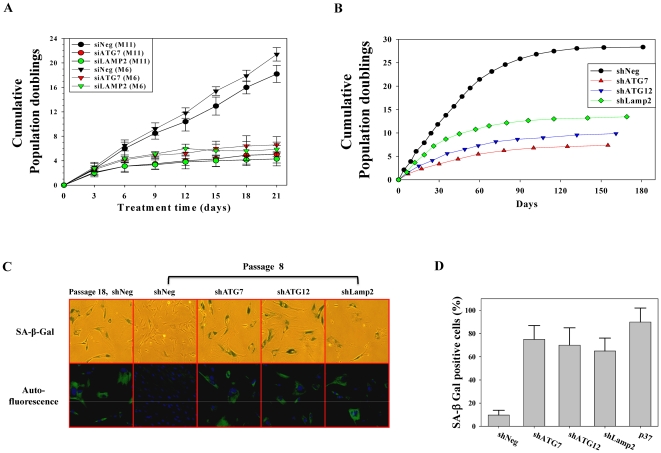
Autophagy impairment induces premature senescence of human fibroblasts. (A) Two strains of primary human fibroblasts were transfected with siRNA to ATG7 or Lamp2 and counted every 3 d for 21 d. Upon confluency, the cells were passaged. Cells transfected with ATG7 and Lamp2 siRNA show reduced proliferation. (B) Stable cell lines expressing shRNA against ATG7, ATG12, and Lamp2 show reduced replicative lifespan. (C) Upper panel: representative micrograph of SA-β-gal staining of stable cell lines at passage 8; Lower panel: representative fluorescence micrograph of autofluorescence of stable cell lines at passage 8. (D) Bars represent the percentage of SA-β-gal positive cells.

**Figure 2:**
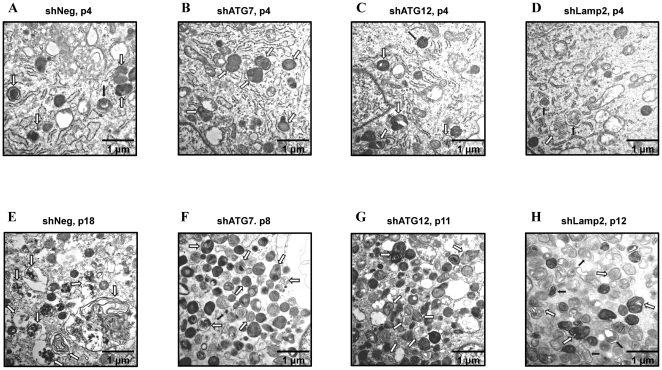
Autophagy impairment-induced prematurely senescent cells show similar morphology as shown in replicatively senescent cells. Electron micrograph of stable cell lines at early passage (A–D) and in a senescent state (E–H). (A and E) control cell line (shNeg); (B and F) ATG7-knockdown cell line (shATG7); (C and G) ATG12-knockdown cell line (shATG12); (D and H) Lamp2-knockdown cell line (shLamp2). Black arrows indicate autophagosome filled with undigested materials (AVi) and white arrows indicate autolysosome filled with partially degraded materials or multi-vesicular/lamellated structures (AVd).

During autophagosome formation, ATG7 is essential for conjugation between ATG5 and ATG12 as well as the lipidation of LC3. The ATG12-ATG5-ATG16 complex and lipidated LC3 contribute to elongation and expansion of autophagosomal membranes. After completion of autophagosome formation, a fusion between the autophagosome and lysosome is required for degradation of engulfed materials. At this fusion step, Lamp2, which is localized at the lysosomal membrane, is critical [24]. To assess the effect of autophagy impairment on senescence, we transfected two primary human fibroblasts (M6 and M11) obtained from two different healthy, young donors with siRNA against ATG7 or Lamp2, which led to growth arrest after 4–6 population doublings (Fig. 1A). These growth-arrested cells showed a senescent phenotype of high SA-β-gal positivity. In addition, autofluorescence, which primarily originates from accumulated lipofuscin [25], and enlarged morphology were observed, which were similar to those seen in replicatively senescent cells (data not shown). We next established human fibroblast cell lines that were stably expressing shRNA against ATG7, ATG12, or Lamp2. Using stable cell lines with reduced expression of the respective genes, we confirmed the senescence-inducing effect of autophagy impairment on replicative lifespan. Cells stably expressing shRNA against ATG7, ATG12, or Lamp2 entered a senescence-like growth-arrested state after 5–7 passages (5–11 population doublings), while cells expressing control shRNA entered senescence after 15 passages (26 population doublings) (Fig. 1A). The stable cell lines that expressed shRNA against ATG7, ATG12, or Lamp2 showed high SA-β-gal positivity (Fig. 1C, D), autofluorescence, and enlarged morphology (Fig. 1C). These results were similar to replicatively senescent cells. Furthermore, transmission electron microscopy showed that autophagy-impaired cell lines at lower passage number exhibited a similar increase of autophagic vacuoles as the replicatively senescent control cell line (Fig. 2E vs. 2F–H), while these cells at very early passage number showed a small number of autophagic vacuoles as young control cell line (Fig.2A vs. 2B–D). In the senescent control cell line, a majority of degenerative autophagic vacuoles (AVd) contained remnants of partially degraded materials, while a minority of AVd showed multi-vesicular or lamellated structures (Fig. 2E). However, in prematurely senescent autophagy-impaired cell lines, the majority of AVd showed multi-vesicular or lamellated structures, while a minority of AVd contained remnants of partially degraded materials (Fig. 2F–H). Accordingly, these data implicate that autophagy impairment can induce premature senescence in primary human fibroblasts.

### Phenotypic characteristics of autophagy impairment-induced prematurely senescent cells

**Figure 3:**
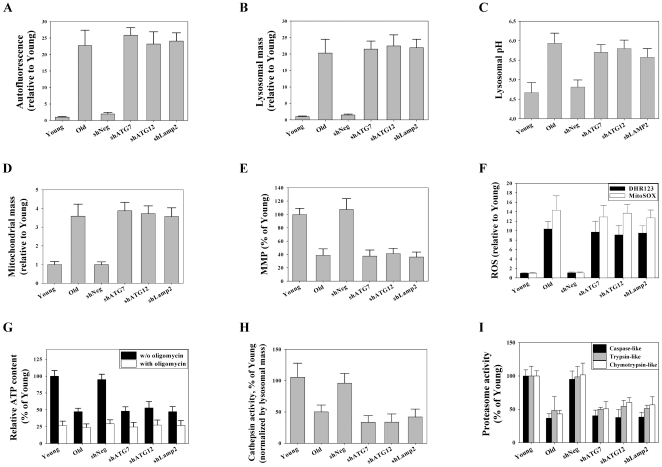
Cells in autophagy impairment-induced premature senescence show the same senescent features as cells in replicative senescence. All experiments were performed on stable cell lines at passage 8. Flow cytometric analysis of autofluorescence (A), lysosomal contents using LytoTracker Red (B), lysosomal pH using FITC-dextran (C), mitochondrial contents using MitoTracker Green FM (D), mitochondrial membrane potential using JC-1 (E), mitochondrial ROS levels using DHR123 and MitoSOX (F), and cathepsin activity using Z-FR-4MβNA as a substrate (H). Luminometric analysis of cellular ATP contents (G) and cell-based proteasome activities (I).

To compare the senescent features of autophagy impairment-induced prematurely senescent cells (hereafter referred to as AIPS cells) with the features of replicatively senescent cells (hereafter referred to as RS cells), the characteristics of AIPS and RS cells were compared. We determined the contents and function of the lysosomes and mitochondria, ROS generation, and proteasomal activity and carried out flow cytometry to quantitate the autofluorescence of the cells. Even at early passages, the stable cell lines with autophagy impairment mediated by RNAi against ATG7, ATG12, or Lamp2 showed increased autofluorescence, mitochondria and lysosome content, and ROS generation, but had decreased mitochondrial membrane potentials, compared to cells expressing control shRNA (Fig. S1). Autofluorescence was increased over 20-fold in both AIPS cells and RS cells (Fig. 3A). A previous report indicated that autophagy impairment would induce mitochondrial dysfunction and ROS generation [26]. Senescent cells exhibit an increase in organellar contents, including lysosomes and mitochondria [27]. AIPS cells showed a high lysosomal and mitochondrial mass similar to RS cells (Fig. 3B and 3D). Senescent cells are known to exhibit a depolarization of the mitochondrial membrane potential [28] and decreased cellular ATP content [29]. AIPS cells showed a decreased mitochondrial membrane potential compared to control cells (Fig. 3E). Moreover, these cells had approximately 50% of the total cellular ATP content compared to the control cells, which was similar to that seen in RS cells (Fig. 3G). Similarly, the glycolytic portion of ATP generation, which was calculated as the oligomycin-insensitive portion of ATP content divided by the total ATP content in each sample, increased from 25% to 50% in both AIPS cells and RS cells compared to the controls.

Senescent cells increase levels of lipofuscin, and the loading of artificial lipofuscin-like molecules on cells hampers lysosomal vacuolar ATPases, which consequently increases the lysosomal pH [30]–[31]. In this regard, we compared the lysosomal pH of AIPS cells and RS cells with control cells. As expected, the lysosomal pH in AIPS cells as well as RS cells was higher than in the control cells (Fig. 3C). In both cases, senescence increased the lysosomal pH by approximately 1 pH unit in the cells.

Senescent cells increase ROS generation. As expected, both AIPS and RS cells had ROS levels approximately 9-fold and 12-fold higher than control cells, respectively, based on DHR123 for hydroxyl radicals and hydrogen peroxides, and MitoSOX for superoxide anions, respectively (Fig. 3F).

**Figure 4:**
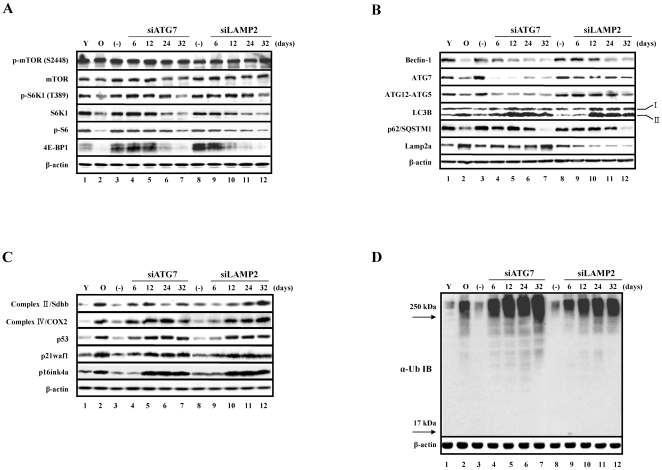
Several factors involved in the mTOR and autophagy pathways are commonly reduced in replicative senescence and premature senescence induced by autophagy impairment. Cells were transfected with siRNA against ATG7 and Lamp2 every 3 d for the indicated times. When the cells were confluent they were passaged. (−) denotes cells transfected with negative siRNA every 3 d for 32 d. At day 24 after transfection with siRNA against ATG7 and Lamp2, the cells showed high SA β-gal activity, as shown in Fig. S1B. (A) Decreased expression of p-S6K1, S6K1, p-S6, and 4E-BP1 in the mTOR pathway. (B) Decreased expression of beclin-1, ATG7, p62/SQSTM1, and ATG12-ATG5 conjugates as well as increased expression of LC3B and Lamp2a in the autophagy pathway. (C) Increased expression of p53, p21waf1, and p16ink4a as well as components of the electron transport chain, Sdhb and COX2. (D) A comparison of the accumulation of poly-ubiquitinated proteins in prematurely senescent cells induced by autophagy impairment and replicative senescent cells. Note that the poly-ubiquitinated proteins with a very high molecular weight (MW) over 250 kD were highly accumulated in prematurely senescent cells induced by autophagy impairment compared to the replicative senescent cells.

Activities of cathepsins, which are major proteolytic enzymes in lysosomes, and proteasomes, are known to be decreased in senescent cells [32]–[33]. The proteasome activities decreased by approximately 50% in both AIPS and RS cells compared to young cells after normalizing the protein contents of the samples (Fig. 3I). In agreement with these results, western blot of the samples using an anti-ubiquitin antibody showed an increase in poly-ubiquitinated proteins in both types of senescent cells. Furthermore, the amounts of poly-ubiquitinated proteins were much higher in AIPS cells than in RS cells (Fig.4D), which was consistent with the observed increases in poly-ubiquitinated proteins *in vitro* and *in vivo* during inhibition of autophagy [34]–[36]. In addition, the cathepsin activity decreased by approximately 50% in both types of senescent cells compared to young cells after normalization of the lysosomal mass (Fig. 3H). Although the overall lysosomal mass increased in senescent cells, the data indicated that at least half of the lysosomes were dysfunctional due to the decreased enzyme activity as a result of the altered pH (Fig. 3B–C, H). In addition, similar results were obtained in AIPS cells when transfected with ATG7 or Lamp2 siRNA, including increases of autofluorescence, organellar contents, and ROS as well as organellar dysfunctions, such as an increase in lysosomal pH and decrease of mitochondrial membrane potential (Fig. S2).

### Down-regulation of mTOR and autophagy pathways in prematurely senescent cells

When components related to the mTOR pathway in both types of senescent cells were monitored, the levels of mTOR or phosphorylated mTOR (p-mTOR) were not markedly changed. However, the levels of S6K1 and phosphorylated S6K1 (p-S6K1), a downstream target of mTOR complex 1 (mTORC1), decreased in both types of senescent cells as well as the level of phosphorylated ribosomal S6 protein (p-S6), which is a downstream target of S6K1 (Fig. 4A). Unexpectedly, 4E-BP1, a repressor of translation initiation factor eIF4E and also a downstream target of mTORC1, was barely expressed in cells undergoing both types of senescence (Fig. 4A). These results suggest that the activity of mTORC1 might decrease in senescent cells, even though the levels of mTOR and p-mTOR were not changed. In addition, protein synthesis, which is one of the cellular functions regulated by mTORC1, decreased by approximately 40% in RS and AIPS cells compared to young cells (data not shown). This observation may have been partially due to the decrease of S6K1.

**Figure 5:**
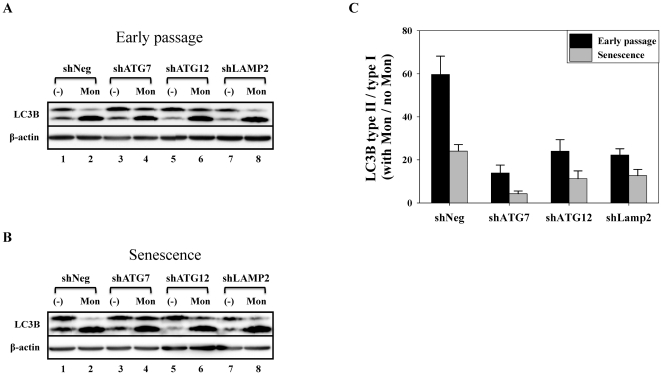
Autophagic flux decreases in HDFs during senescence. At early passage and at senescence, stable cell lines were incubated with or without 40 µM monensin for 2 h. Cells were then collected and used for western blot analysis. (A and B) Representative western blot; (C) Bars represent the level of LC3B type II/type I ratio calculated by normalizing the LC3B type II/type I ratio from monensin-treated samples with LC3B type II/type I ratio from monensin-untreated samples.

Furthermore, the changes in the expression of components related to the autophagy pathway in these cells were monitored. Beclin-1 is a component of the class III PI3 kinase complex and is required for autophagy initiation by various stimuli, such as nutrient starvation, hypoxia, growth factor withdrawal, and rapamycin treatment [24]. The formation of the autophagosome after the initiation of autophagy requires the ubiquitin-like (Ubl) protein system, including LC3 and ATG12, as well as the Ubl-conjugating system, including ATG3 and ATG7. The C-terminal glycine of LC3 is attached to phosphatidylethanolamine (PE), and the C-terminal glycine of ATG12 is attached to ATG5 [37]. The level of beclin-1 was markedly decreased in the cells undergoing both types of senescence. Moreover, the level of beclin-1 gradually decreased in siRNA-transfected cells, which lead to autophagy impairment (Fig. 4B). RNAi-mediated knockdown of ATG7 efficiently decreased ATG12-ATG5 conjugates as well as the expression of ATG7 in cells. In addition, the level of ATG7 was significantly decreased in cells undergoing replicative senescence or premature senescence induced by RNAi-mediated knockdown of Lamp2 (Fig. 4B). ATG12-ATG5 conjugates were also modestly reduced in both types of senescent cells (Fig. 4B), suggesting that the reduction in the levels of ATG7 and ATG12-ATG5 conjugates might be a common phenomenon during cellular senescence. Moreover, the decrease in beclin-1 and ATG7 levels during senescence may be responsible for the reduction of ATG12-ATG5 conjugates. Since SQSTM1/p62 (hereafter referred to as p62) has a ubiquitin-associated domain that is capable of interacting with ubiquitinated proteins, and an LC3-interacting region (LIR)/LC3 recognition sequence (LRS) capable of interacting with LC3, p62 can transport ubiquitinated protein targets to autophagosomes that are destined for degradation [38]. Recently, it was shown that p62 forms SDS-resistant high molecular weight polymers, and therefore p62 monomer is decreased in senescent cells. These polymers are thought to represent inclusion bodies of ubiquitinated protein aggregates [39]. As previously reported, the level of the p62 monomer was markedly decreased in cells undergoing both types of senescence (Fig. 4B). In addition, LC3B, an isoform of LC3, was markedly increased in cells undergoing both types of senescence. The increase of the PE-conjugated form (type II) of LC3B was more noticeable than the increase in the type I of LC3B in AIPS cells (Fig. 4B). This increase of LC3B type II was maximized at day 12 and mildly attenuated at day 32. In addition, the increase of LC3B type II in senescent cells would not be due to the increased autophagic flux, but rather due to the attenuated autophagic flux, since the levels of LC3B type II/type I ratio were more prominently increased in early-passaged cell lines upon inhibition of autophagic flux by the lysosomal inhibitor monensin (Fig. 5). In agreement with the increase in lysosomal mass of cells undergoing both types of senescence (Fig. 3B), the level of Lamp2a, which is a lysosomal membrane protein required for macroautophagy [40] and chaperone-mediated autophagy (CMA) [41], was also markedly increased in these cells (Fig. 4B), and RNAi-mediated knockdown of Lamp2 efficiently decreased the expression of Lamp2a in these cells.

These results suggest that the lowered levels of beclin-1, ATG7, ATG12-ATG5 conjugate, and p62 monomer may be responsible for the reduced autophagy activity observed in senescent cells. In agreement with the observed increase in mitochondrial mass in cells undergoing both types of senescence (Fig. 3D), the levels of succinate dehydrogenase complex subunit B (Sdhb; complex II) and cytochrome c oxidase subunit II (COX2; complex IV) increased markedly in cells undergoing both types of senescence (Fig. 4C). The fluorescent dye staining of lysosomes and mitochondria (Fig. 3B and 3D), and the immunoblot of Lamp2a in the lysosome and Sdhb and COX2 in the mitochondria (Fig. 4B–C), all showed that the organellar contents in senescent cells were indeed increased compared to control. However, these organelles had clear functional defects, as shown by activity analyses of the lysosomes (Fig. 3C and 3H) and mitochondria (Fig. 3E and 3G).

### Autophagy impairment-induced premature senescence is ROS-dependent and p53-dependent

**Figure 6:**
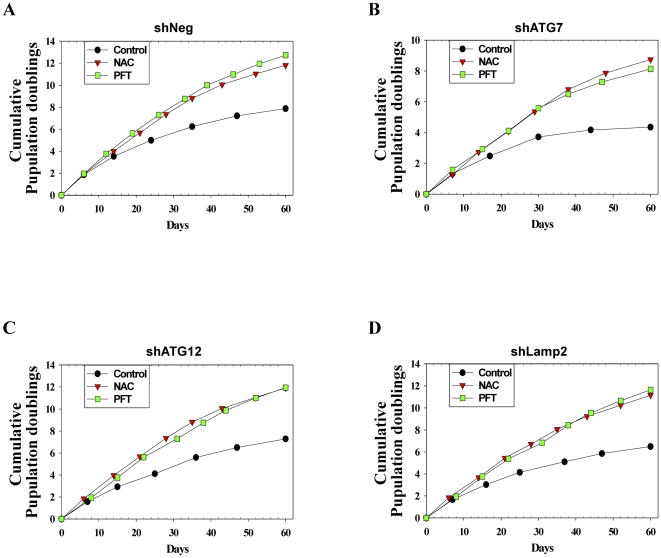
NAC and pifithrin-α (PFT) delay the premature senescence induced by autophagy impairment. 5 mM NAC or 5 µM PFT was administered every 3 d to stable cell lines from a population doubling time of 6 d. Upon confluency, cells were passaged and counted for 2 months.

**Figure 7:**
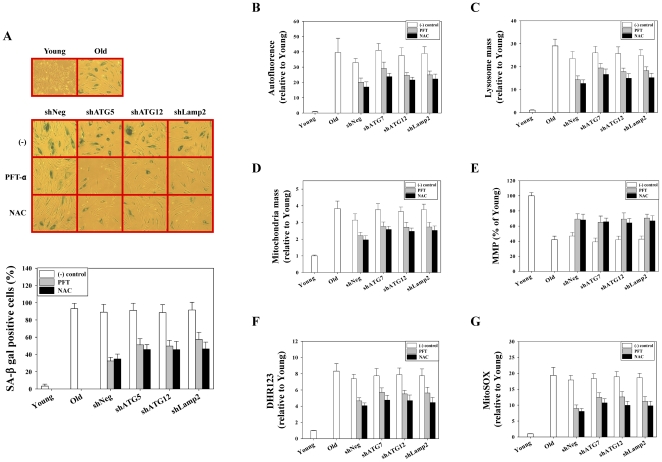
NAC and pifithrin-α (PFT) delay the expression of senescence phenotypes in prematurely senescent cells induced by autophagy impairment. Stable cell lines were cultured as shown in Fig. 6. Two months after drug treatment, the cells were assayed for the expression of SA-β-gal activity. Cell lines cultured without drugs showed high SA-β-gal activity. These cells were assayed for determination of expression levels of senescence phenotypes. (A) Left panel: Representative micrograph of SA-β-gal staining in stable cell lines cultured with or without NAC and PFT; Right panel: Bars represent the percentage of SA-β-gal positive cells. Flow cytometric analysis of autofluorescence (B), lysosomal content using LytoTracker Red (C), mitochondrial content using MitoTracker Green FM (D), mitochondrial membrane potential using JC-1 (E), and mitochondrial ROS levels using DHR123 (F) and MitoSOX (G) in stable cell lines cultured with or without NAC and PFT.

The increased ROS generation, which mainly originates from mitochondrial dysfunction, can activate the p53 tumor suppressor pathway and result in premature senescence [42]. In our study, the stable cell lines with autophagy impairment showed mitochondrial dysfunction (decreased mitochondrial membrane potential) with the increased mitochondrial ROS generation at the early passage compared to control cells (Fig. S1C-D). Accordingly, we performed experiments to determine the localization of p53 protein. At early passage, the immunofluorescence of p53 in autophagy-impaired cell lines showed a moderate signal of p53, which was mainly localized in nuclei, while the control cell line showed a very faint p53 signal in the nuclei. As expected, however, during senescence, all cell lines including the AIPS and RS cells showed a strong p53 signal in nuclei (data not shown). In addition, the levels of p53, its downstream target p21waf1, and the CDK inhibitor p16ink4a were all increased in AIPS cells as well as in RS cells (Fig. 4C). The levels of p53, p21waf1, and p16ink4a reached a maximum level at day 12 and remained at that level in the AIPS cells. Stress-induced p53 activation might induce apoptosis or autophagy through the transcriptional induction of DRAM, PUMA, or NOXA [43]. However, p53 induced by autophagy impairment did not activate the expression of DRAM, PUMA, or NOXA (data not shown). Therefore, although p53 is activated through autophagy impairment, we excluded the possibility that apoptosis or autophagy regulated by p53 induction affects premature senescence through autophagy impairment. Given that autophagy-impaired cell lines produced more ROS compared to the control cell line, it is anticipated that autophagy impairment would stress the cells by the increased ROS generation, and that oxidative stress-mediated p53 activation would force the cells into premature senescence. Therefore, we examined whether the inhibition of p53 or scavenging of ROS would be effective in delaying premature senescence induced by autophagy impairment. Pifithrin-α (PFT), a chemical inhibitor of p53 [44], and N-acetyl cysteine (NAC), an antioxidant, were administered every 3 d to the control cell lines and stable cell lines of autophagy impairment from a population doubling time of 6 d in order to inhibit p53 and ROS scavenging, respectively. When NAC or PFT were administered to the cells, the replicative lifespan increased by approximately 4–5 population doublings (Fig. 6). In addition, knockdown of p53 using siRNA increased the cellular lifespan of the cell lines by approximately 2–4 population doublings for 30 d (data not shown). Two months after the drug treatment, the cells were assayed for SA-β-gal activity. As expected, almost all of the cells in the untreated group, which had a population doubling time over 18 d, stained positive for SA-β-gal activity. However, approximately half of the cells in the PFT- or NAC-treated groups, which had a population doubling time below 9 d, stained positive for SA-β-gal activity (Fig. 7A).

The senescent phenotypes were compared between the control and PFT- or NAC-treated cell lines. The increase in autofluorescence and organellar contents (lysosome and mitochondria) was markedly reduced in PFT- or NAC-treated cell lines compared to control cell lines (Fig. 7B-D). In addition, the increase in mitochondrial ROS was markedly decreased in drug-treated cell lines compared to control cell lines (Fig. 7F–G). Furthermore, depolarization of the mitochondrial membrane potential was markedly attenuated in PFT- or NAC-treated cell lines compared to the control (Fig. 7E). These data implicate that premature senescence induced by autophagy impairment can be delayed morphologically and functionally by inhibiting p53 activation or ROS scavenging (Fig. 6 and Fig. 7).

**Figure 8:**
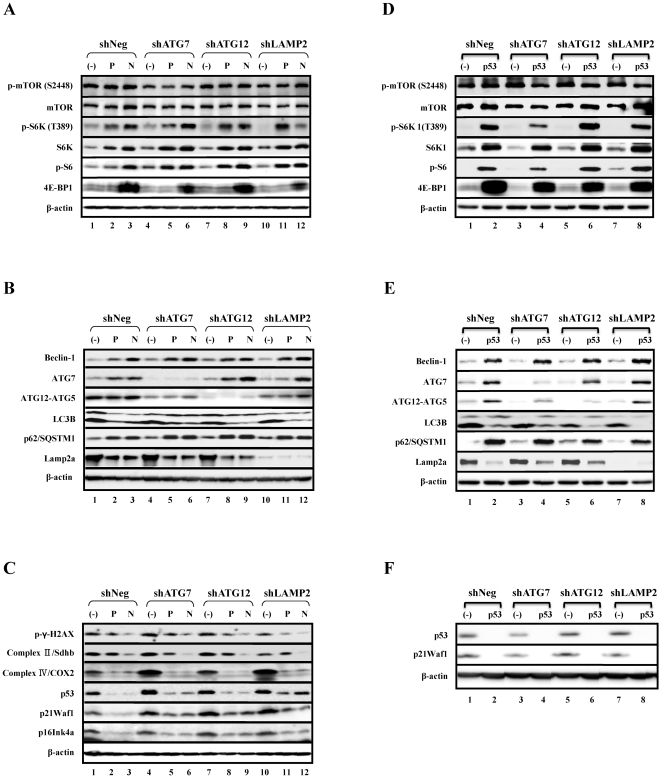
NAC, PFT, or knockdown of p53 using siRNA restore the expression of genes in the mTOR and autophagy pathways that are reduced during premature senescence. (A–C) Stable cell lines were cultured as shown in Fig. 5. (D–F) Stable cell lines were transfected with negative siRNA and siRNA to p53 every 3 d for 5 weeks. P and N denote PFT and NAC, respectively, and (−) and p53 denote negative siRNA and siRNA to p53, respectively. (A and D) Restoration of expression levels of p-S6K1, S6K, p-S6P, and 4E-BP1 in the mTOR pathway by PFT and NAC treatment, and by knockdown of p53. (B and E) Restoration of expression levels of beclin-1, ATG7, and p62/SQSTM1 as well as decreased expression levels of LC3B and Lamp2a in the autophagy pathway by PFT and NAC treatment as well as knockdown of p53. (C and F) Decreased expression levels of p53, p21waf1, p16ink4a, and γ-H2AX as well as components of the electron transport chains, Sdhb and COX2, by PFT and NAC treatment, and decreased expression levels of p53 and p21waf1 by knockdown of p53.

We next examined the changes in expression of components related to mTOR pathway, autophagy, and p53 pathways in PFT- and NAC-treated cell lines as well as p53-knockdown cell lines compared to the control. The levels of mTOR or p-mTOR were not significantly altered in PFT- or NAC-treated cell lines, or p53-knockdown cell lines compared to the control (Fig. 8A and 8D). However, the levels of S6K1, p-S6K1, p-S6, beclin-1, ATG7, and p62 monomer were restored in PFT- and NAC-treated cell lines as well as p53-knockdown cell lines compared to the control cell lines (Fig. 8A, B, D, E). However, 4E-BP1 was restored in the NAC-treated cell lines and p53-knockdown cell lines, but not in the PFT-treated cell lines (Fig. 5A). In contrast, the level of ATG12-ATG5 conjugates was not changed in PFT- or NAC-treated cell lines, whereas ATG12-ATG5 conjugates were restored in p53-knockdown cell lines (Fig. 8B and 8E). Moreover, the factors that were increased in cells undergoing both types of senescence, including the type II form of LC3B, Lamp2a, COX2, p53, p21waf1, and p16ink4a, were markedly reduced in PFT- and NAC-treated cell lines as well as p53-knockdown cell lines compared to control cells (Fig. 8B-C and 8E-F). The level of Sdhb was markedly reduced in NAC-treated but not PFT-treated cell lines compared to control cells (Fig. 8C). In addition, the level of the well-known DNA damage marker, γ-H2AX, was reduced in PFT- or NAC-treated cell lines compared to controls (Fig. 8C).Taken together, the expression levels of components related to mTOR pathway, autophagy pathway, and p53 and p21waf1 were all similarly restored in NAC- or pifithrin-α-treated cells as well as in p53 knockdown experiments.

Overall, ROS scavenging by NAC was slightly more effective than PFT-mediated p53 inhibition in the restoration of protein expression in AIPS cells (Fig. 8A–C). Moreover, the stable cell lines of autophagy impairment had already exhibited mitochondrial dysfunction and increased mitochondrial ROS generation at early passages compared to control cells (Fig. S1). Therefore, these data suggest that the activation of p53, which presumably occurred as a result of the increased ROS generation originating from dysfunctional mitochondria, might have an important role in the premature senescence induced by autophagy impairment.

## Discussion

In this study, we have demonstrated that autophagy impairment induces premature senescence in human primary fibroblasts. In addition, we showed that several important components that have been implicated in the mTOR pathway (S6K1, p-S6, and 4E-BP1) or autophagy pathway (Beclin-1, ATG7, p62 monomer, and ATG12-ATG5 conjugates) are down-regulated in both RS cells and AIPS cells. Furthermore, we have shown that p53 inhibition or ROS scavenging can adjust premature senescence, induced by autophagy impairment, and can also reverse the changes seen in the mTOR and autophagy pathways. Therefore, our findings suggest that autophagy impairment can induce premature senescence in human primary fibroblasts through activation of the p53 tumor suppressor pathway due to increased ROS generation from an accumulation of dysfunctional mitochondria, which are usually eliminated by autophagic degradation.

Autophagy is a catabolic process by which cytoplasmic components, including macromolecules and organelles, are degraded. Autophagy plays a role in the physiology of several cellular processes, pathogenesis of several disease processes, and health [1]. Many studies have shown that autophagy activation can prolong the lifespan of organisms from yeast to flies [2]–[6] and that autophagy activation is important for lifespan extension [5], [9]–[11]. Moreover, a conditional knockout of ATG7 in mice, which is an essential gene for autophagy, caused pathological defects in the liver [34], neuro-degeneration, and early death [35]. These studies suggest that autophagy-activating interventions might be useful tools for promoting longevity. On the other hand, it has been reported that overexpression of ULK3, an isoform of ULK1, induces autophagy activation and reduces replicative lifespan of human fibroblasts by approximately 20% [23]. Therefore, a bell-shaped relationship between autophagy activity and cell survival has been proposed. The maintenance of physiological levels of autophagy would be essential for normal cellular homeostasis, while a deficiency or excessive autophagy might be unfavorable [45]. Interestingly, ULK3, but not ULK1, has been reported to be involved in the sonic hedgehog signaling pathway [46], suggesting that the excessive autophagy by either the overexpression of ULK3 or an as yet unidentified function might affect replicative lifespan. Therefore, it remains to be determined whether excessive autophagy induced by the overexpression of genes other than ULK3 can still reduce replicative lifespan. In this aspect, we have shown in this study that autophagy impairment induces premature senescence in a p53-dependent and ROS-dependent manner.

To our knowledge, this is the first report demonstrating that the protein levels of S6K1 and 4E-BP1 (mTOR pathway) as well as Beclin-1 and ATG7 (autophagy pathway) are decreased in senescent cells. Phosphorylations of S6K1 at Thr421/Ser424 and at Thr389 were diminished in replicatively senescent fibroblasts, and phosphorylation of ribosomal S6 protein was severely reduced in these cells [47]. Thus, the decrease of protein synthesis might be related to the diminished levels of the proteins and phosphorylation status of S6K1 and S6 protein, even though the levels of mTOR and p-mTOR were unchanged. Although the mechanism by which the different forms of autophagy decrease with aging is as yet unclear, it is well accepted that the functional decline of autophagy is involved in the aging process, and the prevention of this decline should have a beneficial effect on lifespan [48]–[49]. Recently, chaperone-mediated autophagy (CMA) has been reported to decrease with aging due to a reduction of the lysosomal receptor for CMA. In addition, the restoration of CMA function with a knock-in of the lysosomal receptor for CMA in the liver results in an improvement of hepatic function in aging animals [50]. In contrast to CMA, the mechanism by which macroautophagy, another form of autophagy, decreases with aging is not yet known. In this study, we demonstrated that Beclin-1 and ATG7 levels are decreased in senescent cells, suggesting that the initiation step of autophagy as well as the elongation step of the autophagosome might be damaged in senescent cells as previously reported [51]. In addition, the decline of the p62 monomer, which is required for the transport of poly-ubiquitinated proteins into the autophagosome, may induce defective clearance and accumulation of poly-ubiquitinated proteins in cells as previously reported [39]. Therefore, our study has shown that autophagy in senescent cells is damaged, at least in part, at multiple steps of the pathway, including autophagic initiation, autophagosome formation, and the degradation of poly-ubiquitinated proteins.

In conclusion, our findings indicate that autophagy impairment can induce premature senescence in human fibroblasts through the activation of p53 by increased ROS generation originating from dysfunctional mitochondria. Moreover, our study has shown that during senescence, the protein levels of S6K1, 4E-BP1, beclin-1, and ATG7 are decreased. Further studies are needed to test whether restoration of the factors of the autophagy pathway can improve the autophagic function of senescent cells, which would result in a delay of the cellular aging process and final extension of cellular lifespan.

## Materials and Methods

### Cell cultures

Normal human diploid fibroblasts (HDFs) were obtained from the foreskin of an 11 year-old donor (M11 strain) and 6 year-old donor (M6 strain) as described previously [52]. HDFs were cultured in Dulbecco's modified Eagle's medium (DMEM) containing 25 mM glucose supplemented with 10% fetal bovine serum (FBS), 100 units/ml penicillin, and 100 ug/ml streptomycin. When confluent, the cells were split 1∶4 during early passages and 1∶2 during late passages. The culture medium was changed every 4 d. The number of population doublings (PDs) (*n*) was calculated using the equation: *n* = log_2_ *F/I*, where *F* and *I* are the numbers of cells at the end and those seeded at the beginning of one passage, respectively. When the population doubling time of the cells was over 21 d, the cells were considered to be senescent. Cellular senescence was confirmed by the expression of SA-ß-gal activity. For transfection of siRNA, the cells were seeded at a 1/4 dilution in the culture dish 3-4 d before the transfection. After culturing the cells overnight (O/N), the siRNAs were transfected with Lipofectamine RNAiMAX (Invitrogen) according to the manufacturer's protocol. When the cells were confluent, they were counted, passaged, and transfected with siRNA every 3 d for 24 d. All siRNAs were purchased from Bioneer. The siRNA sequences were as follows: ATG7, sense strand, CAG CUA UUG GAA CAC UGU A, LAMP2, sense strand, GAA AAU GCC ACU UGC CUU U, p53, sense strand, CAC UAC AAC UAC AUG UGU A and the negative control, sense strand, CCU ACG CCA CCA AUU UCG U. To establish stable cell lines, the cells (M11 strain) were infected with Lentiviruses expressing shRNA to ATG7, ATG12, or LAMP2 (Santa Cruz) at an early passage and were selected with 2 µg/ml puromycin (Sigma) for 7 d. When confluent, the cells were trypsinized and split by 1∶4. This time point was designated as PD 0. Pooled clones were passaged until they reached senescence. To determine autophagic flux, early-passaged (at passage 3) cell lines and senescent (population doubling time over 15 d) cell lines were incubated with or without 40 µM monensin (Sigma) for 2 h.

### SA-β-Gal staining

SA-β-Gal staining was carried out as previously described by Dimri et al. (1995). The cells were stained after fixation in 3% formaldehyde for 5 min with freshly prepared SA-β-Gal staining solution overnight at 37°C. The number of SA-β-Gal-positive cells in randomly-selected fields was expressed as a percentage of all cells counted.

### Western blot analysis

The cells were lysed with RIPA buffer (50 mM Tris-Cl, pH 7.5, 150 mM NaCl, 1% Nonidet P-40, 0.5% sodium deoxycholate, and 0.1% SDS) supplemented with a protease inhibitor mixture (Sigma), sodium fluoride, and sodium orthovanadate (Sigma). After 20 min incubation on ice, the cell lysates were collected after centrifugation. The protein concentration in the lysates was determined using the BCA protein assay reagent (Bio-Rad). Approximately 10 µg of protein were separated by SDS-PAGE and transferred to a nitrocellulose membrane (Millipore). The blots were then incubated with antibodies against p-mTOR (S2448), mTOR, p-S6K (T389), S6K, p-S6 protein, 4E-BP1, Beclin-1, ATG7, ATG12, LC3B (Cell Signaling), p62/SQSTM1, p53, p21waf1, Lamp2a, ubiquitin (Santa Cruz), γ-H2AX, p16ink4a (Pharmingen), Sdhb, COX2 (MitoScience), and β-actin (Sigma). The protein bands were visualized by using horseradish peroxidase-conjugated secondary antibodies and SuperSignal West Pico or Femto substrate (Thermo Scientific).

### Measurement of autofluorescence, ROS, organellar contents, and mitochondrial membrane potential

For quantitation of autofluorescence, the cells were washed with PBS, trypsinized, collected in PBS, and analyzed on a FACSCaliber (Beckton Dickson). For quantitation of mitochondrial ROS, the cells were incubated with 5 µM DHR123 (Anaspec) and 0.2 µM MitoSOX (Invitrogen) for 30 min at 37°C, washed with PBS, trypsinized, collected in PBS, and analyzed on a FACSCaliber (Beckton Dickson). For quantitation of organellar contents, the cells were incubated with 60 nM MitoTracker Green FM and 50 nM LysoTracker Red (Invitrogen) for 30 min at 37°C, washed with PBS, trypsinized, collected in PBS, and analyzed on a FACSCaliber (Beckton Dickson). For measurement of the mitochondrial membrane potential, the cells were incubated with 0.3 µg**/**ml JC-1 (Invitrogen) for 30 min at 37°C and prepared for FACS analysis as previously described [53]. The results were analyzed by using Cell Quest 3.2 software (Beckton Dickson) for analysis.

### Measurement of lysosomal pH

The lysosomal pH was measured by flow cytometry as previously described [54]–[55]. The cells were incubated in normal medium containing 3 mg/ml FITC-dextran (150 kD; Polysciences) for 3 d, washed with PBS, and subsequently incubated in normal medium for 24 h. Cells were washed with PBS, trypsinized, collected in PBS, and analyzed on a FACSCaliber (Beckton Dickson). The excitation wavelength was 488 nm and the emission fluorescence for FITC was monitored at 530 nm (FL-1) and 610 nm (FL-2). The FL-1/FL-2 ratio of the samples was converted to pH using a standard curve from cells loaded with FITC-dextran. For construction of a standard curve, the cells that were loaded with FITC-dextran were prepared for FACS analysis. Prior to analysis, the cells were incubated in phosphate-citrate buffers (pH 4.0–7.0) containing 50 mM NaN_3_, 50 mM 2-deoxyglucose, and 10 µM Nigericin for 20 min. The background autofluorescence from control cells that were incubated in normal medium without FITC-dextran was subtracted from the fluorescence of cells that were incubated with FITC-dextran.

### Measurement of cathepsin activity

The cathepsin activity was measured by flow cytometry as previously described [56]–[57]. The cells were incubated with or without leupeptin and 10 µM E-64d (Calbiochem) for 30 min and were prepared for FACS analysis. Prior to analysis, the cells were resuspended in serum-free and phenol red-free DMEM containing 50 µM 5-nitrosalicylaldehyde (5-NSA) with or without leupeptin and 10 µM E-64d. The cells were then incubated for 2 h at 37°C with 0.2 mM Z-Phe-Arg-4-methoxy-β-naphthylamine (Z-FR-4MβNA). The cells were washed with PBS 3 times and the fluorescence was monitored at excitation 488 nm/emission 530 nm. The background autofluorescence from cells incubated with only 5-NSA was subtracted from the fluorescence of cells incubated with Z-FR-4MβNA. The specific activity of cathepsin was calculated by subtracting the fluorescence of cells with inhibitors from the fluorescence of cells without inhibitors.

### Measurement of cellular ATP levels

The cells were incubated with or without 8 µg/ml oligomycin (Sigma) for 24 h, lysed with lysis buffer, and the ATP content was measured using the ViaLight Plus Kit (Lonza) according to the manufacturer's instructions. For measurements of relative ATP content, the luminescence of each sample was normalized with the protein content in each sample.

### Measurement of cell-based proteasome activity

The cells were incubated with or without proteasome inhibitors (BioMol) for 24 h, lysed with lysis buffer, and the proteasome activity was measured using the Proteasome-Glo cell-based assay Kit (Promega) according to the manufacturer's instructions. To measure the relative proteasome activity, the luminescence of each sample was normalized with the protein content in each sample.

### Electron microscopy (EM)

Early passaged cell lines and senescent cell lines were trypsinized, washed with PBS, and fixed in 2% glutaraldehyde containing 2% paraformaldehyde in 30 mM phosphate buffer (pH 7.4) for 2 h at RT. Cells were washed with 0.2 M HEPES (pH 7.4) and 0.1 M phosphate buffer (pH 7.4), consecutively. Cells were then post-fixed in 1% OsO_4_ for 2 h at RT. After dehydration in graded series of ethanol, the cells were embedded in Epon 812 (Fluka) and polymerized at 60°C for 2 d. Thin sections were cut on a Leica EM UC6 and then counterstained with uranyl acetate and lead citrate. EM images were acquired from thin sections using a JEOL JEM-1400 transmission electron microscope equipped with Orius SC1000 digital camera (Gatan, Warrendale, U.S.A.).

## Supporting Information

Figure S1 **Stable cell lines with autophagy impairment show increased ROS at early passages.** At passage 2, stable cell lines were used for flow cytometric analysis of autofluorescence (A), lysosomal contents using LytoTracker Red and mitochondrial contents using MitoTracker Green FM (B), mitochondrial membrane potential using JC-1 (C), and mitochondrial ROS levels using DHR123 and MitoSOX (D).
(TIFF)

Figure S2 **Premature senescence induced by autophagy impairment using siRNA show the same senescent features as replicative senescence.** Cells transfected with siRNA every 3 d for 24 d were used for flow cytometric analysis of autofluorescence (A), lysosomal contents using LytoTracker Red (B), lysosomal pH using FITC-dextran (C), mitochondrial contents using MitoTracker Green FM (D), mitochondrial membrane potential using JC-1 (E), and mitochondrial ROS levels using DHR123 and MitoSOX (F).
(TIFF)
