# Supplementary figures and images for: Autophagy Impairment Induces Premature Senescence in Primary Human Fibroblasts
Source: PLoS One. 2011 Aug 8;6(8):e23367. doi: 10.1371/journal.pone.0023367 (PMC3152578; doi:10.1371/journal.pone.0023367)

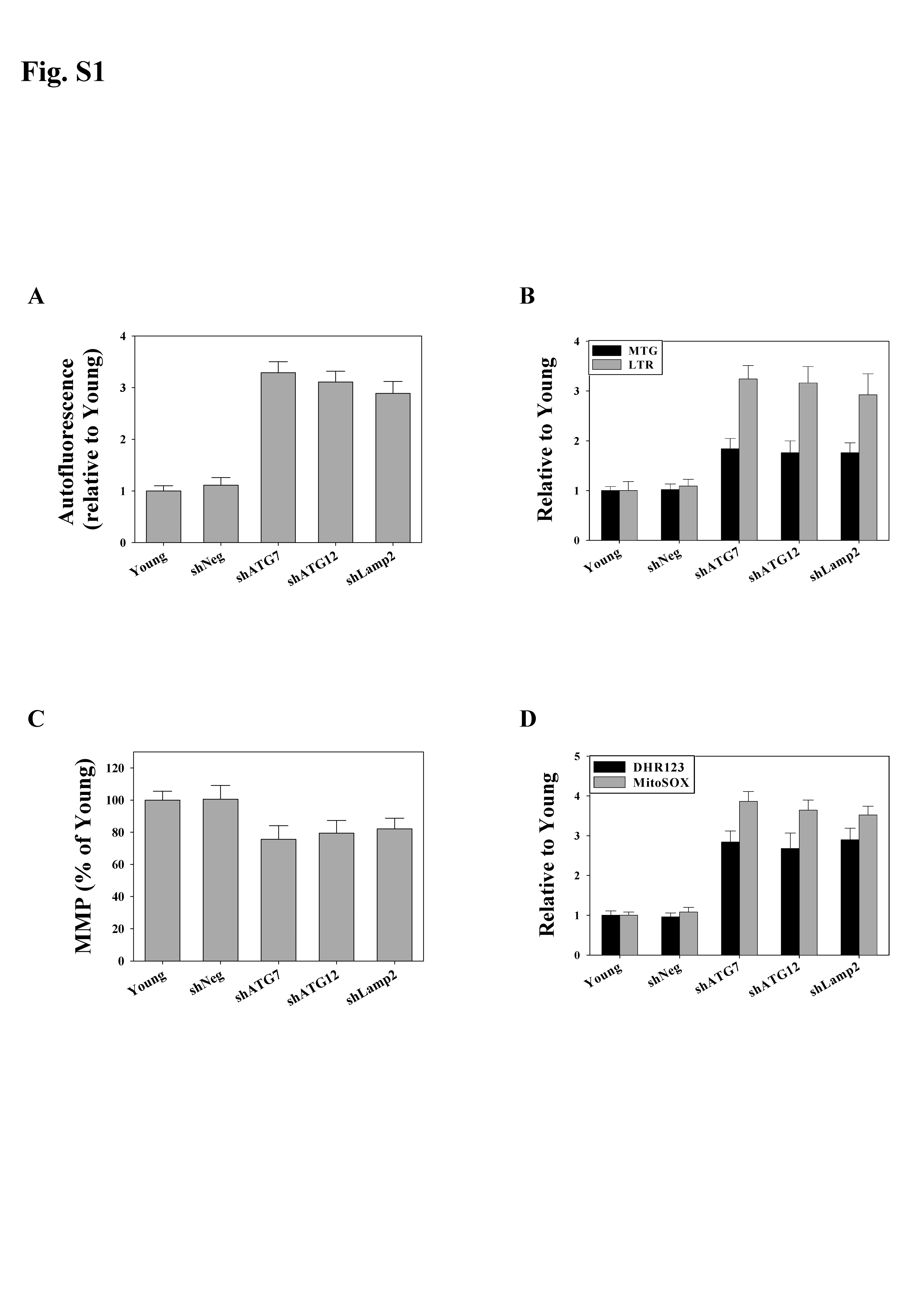

Supplement: Figure S1 — Stable cell lines with autophagy impairment show increased ROS at early passages. At passage 2, stable cell lines were used for flow cytometric analysis of autofluorescence (A), lysosomal contents using LytoTracker Red and mitochondrial contents using MitoTracker Green FM (B), mitochondrial membrane potential using JC-1 (C), and mitochondrial ROS levels using DHR123 and MitoSOX (D). (TIFF) [file pone.0023367.s001.tif]

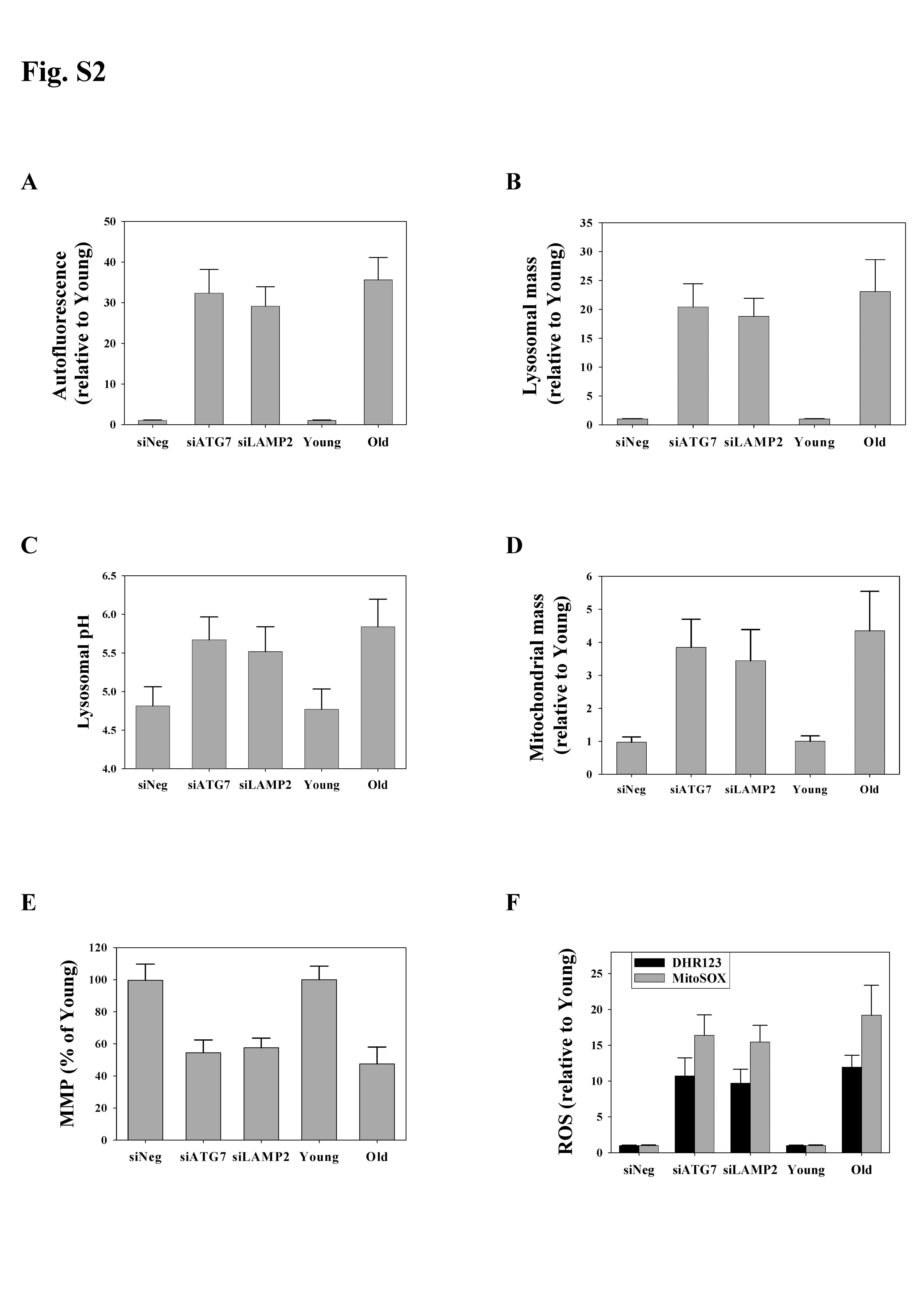

Supplement: Figure S2 — Premature senescence induced by autophagy impairment using siRNA show the same senescent features as replicative senescence. Cells transfected with siRNA every 3 d for 24 d were used for flow cytometric analysis of autofluorescence (A), lysosomal contents using LytoTracker Red (B), lysosomal pH using FITC-dextran (C), mitochondrial contents using MitoTracker Green FM (D), mitochondrial membrane potential using JC-1 (E), and mitochondrial ROS levels using DHR123 and MitoSOX (F). (TIFF) [file pone.0023367.s002.tif]
